# Linking leaf embolism resistance with pit membrane characteristics

**DOI:** 10.1093/plphys/kiac293

**Published:** 2022-06-15

**Authors:** Amanda A Cardoso

**Affiliations:** Department of Crop and Soil Sciences, North Carolina State University, Raleigh, North Carolina 27695, USA

**Figure 1:**
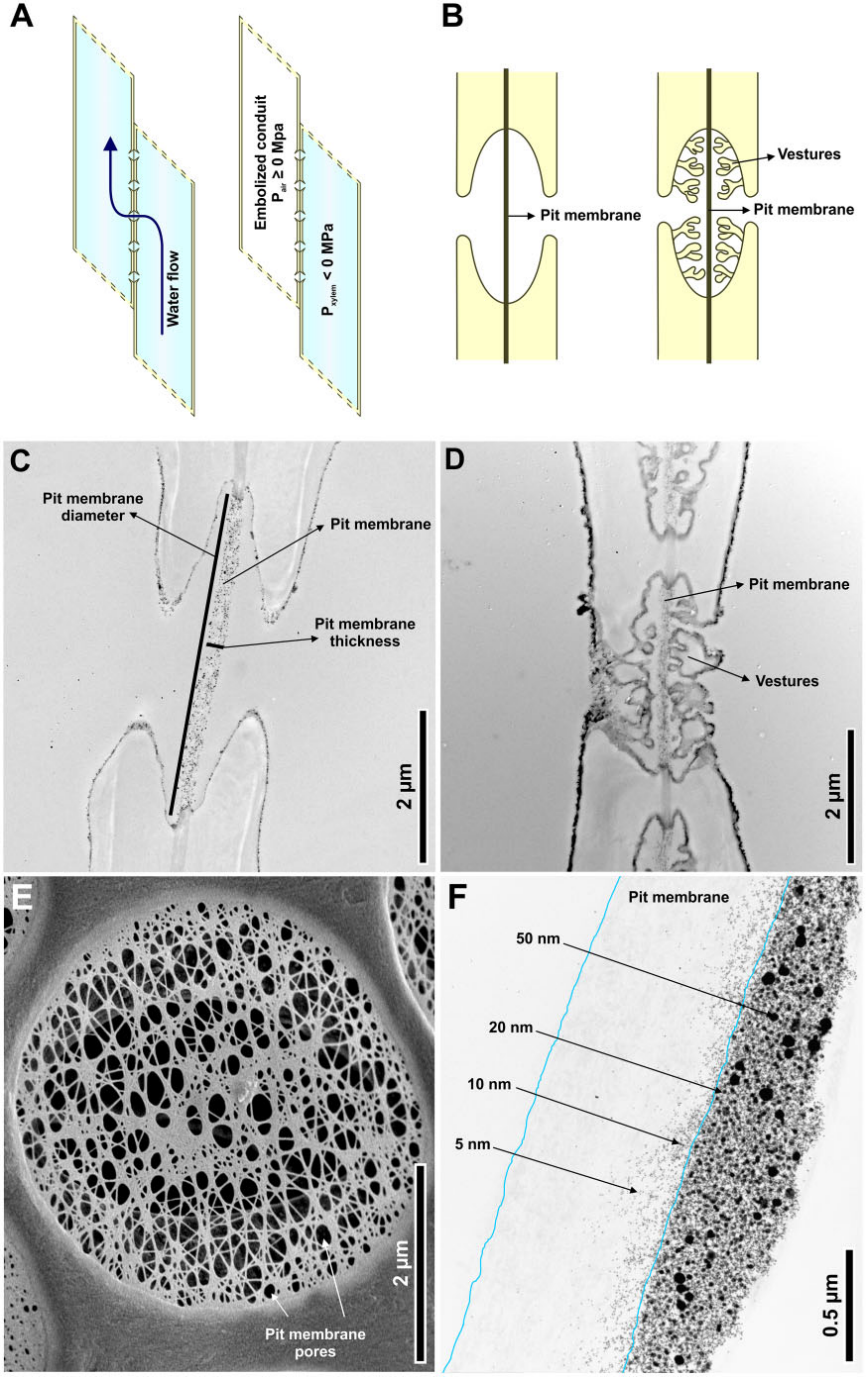
Structure of interconduit pits and pit membranes. A, Adjacent xylem conduits connected by interconduit pits. On the left, both conduits are sap-filled and functional. On the right, one conduit is gas-filled (embolized) so that air pressure (*P*_air_) is ≥0 MPa and the other conduit is sap-filled so that xylem pressure (*P*_xylem_) is <0 MPa. B, Representation of non-vestured and vestured interconduit pits with pit membranes. C, TEM image of non-vestured interconduit pit in sycamore maple (*Acer pseudoplatanus*). D, TEM image of a typical vestured interconduit pit in *Tachigali melinonii*. E, Scanning electron microscope (SEM) image of hazelnut (*Corylus avellana*) showing a “single layer” of a pit membrane and large, artificial pores, which does not represent an intact pit membrane under natural conditions. F, TEM image showing penetration of gold particles of different sizes into a pit membrane (outlined by blue lines) of camphor tree (*Cinnamomum camphora*), that is, a method used by Levionnois et al. (2022) to estimate pore constriction size. The perfusion direction of 5, 10, 20, and 50 nm particles was from right to left, with small particles going deeper into the membrane than larger ones. TEM and SEM images are courtesy of Prof. Dr. Steven Jansen (Ulm University).

Long-distance transport of water is essential for plant function and this process is largely facilitated by the xylem. Water is transported under negative pressure within xylem conduits (i.e. unicellular tracheids and multicellular vessels) and between adjacent conduits through the pit membranes of interconduit pits (Figure 1). In some species, pit apertures are lined with branched or irregularly shaped protuberances called vestures and such pits are referred to as vestured pits. In angiosperms, pit membranes are homogeneous, modified primary cell walls consisting of microfibril layers that form a three-dimensional porous matrix (Kaack et al., 2019). This matrix is composed of solid material (i.e. cellulose) forming numerous geometrically irregular pore spaces that are interconnected through pore constrictions of varying sizes. Such constrictions strongly influence the hydraulic resistance of fluid transport across the pit membrane (Figure 1) (for a three-dimensional representation of pit membranes, pore spaces, and pore constrictions, see Kaack et al. [2019] and Zhang et al. [2020]).

During soil drought, increasing negative pressure of the xylem above a threshold results in the movement of gas from neighboring gas-filled conduits into the lumen of sap-filled conduits through pit membrane pores. Gas entry into a sap-filled conduit increases the likelihood that a large bubble is formed, which can block water transport within that conduit (embolism), thereby reducing hydraulic conductances (Kaack et al., 2021). The xylem vulnerability to embolism of several plant organs can be quantified by vulnerability curves that describe the accumulation of embolisms as a function of water potential. The water potential at which 50% of the xylem is embolized (*P*_50_) represents the xylem resistance to embolism of that particular organ and species (Choat et al., 2012).

Leaf embolism results in declines in leaf function and, ultimately, can cause leaf mortality (Cardoso et al., 2020; Brodribb et al., 2021). Such detrimental consequences demonstrate the importance of leaf embolism resistance to whole-plant drought tolerance. Yet, unlike the case with stems, the structural determinants of embolism resistance in leaves remain largely unknown. In this issue of *Plant Physiology*, Levionnois et al. (2022) assessed the linkage between leaf embolism resistance and pit membrane characteristics across Neotropical tree species, providing valuable insights into the xylem embolism resistance of leaves.

One of the most interesting findings of this study is that the maximum pit membrane thickness (*T*_PM_) was highly predictive of leaf embolism resistance (represented by the *P*_50_), with thick pit membranes being associated with high leaf embolism resistance. This was true across non-vestured species at the individual level (when these two parameters were collected for adjacent leaves of the same shoot). Similar associations have been previously demonstrated at the stem level across different biomes, plant forms, and species. The mechanistic explanation for this association relies on the hypothesis that thick pit membranes have higher chances of presenting narrow pore constrictions because thick pit membranes can be considered as consisting of more cellulose layers and thus more pore constrictions than thin pit membranes (Kaack et al., 2021).

The association between these two traits, however, did not hold true at the species level (i.e. when mean *P*_50_ was obtained from different individuals and *T*_PM_ was obtained for a single individual per species). A possible explanation might be linked to the large intra-specific variation in leaf *P*_50_ observed for several species in this study (for the vulnerability curves of all species, see Supplementary Material of Levionnois et al. [2020]). It is possible (or even likely) that, like leaf *P*_50_, *T*_PM_ might also exhibit a large variability across different leaves, especially from different individuals, yielding a low agreement between mean leaf *P*_50_ and the *T*_PM_ obtained for a single leaf. Although variations in pit membrane between species have been relatively well characterized, variations within species are largely unknown. This occurs as researchers often assess only one sample per species, because of the time-consuming nature of transmission electron microscopy (TEM). However, pit membranes may decline in thickness and become more compact over the course of a growing season (Schmid and Machado, 1968; Sorek et al., 2021). Additional changes, such as pit membrane aspiration and deformation by fluid pressure and/or flow conditions, can also induce pit membrane shrinkage in an irreversible way, thus influencing pit membrane dimensions of that particular sample.

Unlike *T*_PM_, Levionnois et al. (2022) found pit membrane diameter (*D*_PM_) and the ratio between maximum pit membrane thickness to pit membrane diameter (*T*_PM_/*D*_PM_) to be well correlated with *P*_50_ at the species level for non-vestured pit species. This may suggest that, unlike *T*_PM_, *D*_PM_ and *T*_PM_/*D*_PM_ might not exhibit a large variation within species. Therefore, close relationships between mean *P*_50_ (obtained from several individuals) and a single point of *D*_PM_ or a single point of *T*_PM_/*D*_PM_ (representing the mean value for the species) remain possible. Although our mechanistic understanding of the relationships between leaf *P*_50_ versus *D*_PM_ or *T*_PM_/*D*_PM_ remain unclear, the authors speculate that smaller pit membranes (with lower *D*_PM_) are more resistant to stretching and deflection for a given pressure difference, potentially avoiding local increases in pore constriction sizes associated with membrane stretching. In agreement with a stretching-based mechanism of embolism spreading, species with higher *T*_PM_/*D*_PM_ were also more resistant to leaf embolism, which suggests that *T*_PM_/*D*_PM_ also reflects pit membrane stiffness: thick, small pit membranes are stiffer and thus more resistant against stretching for a given pressure difference. It is important to mention, however, that angiosperm pit membrane stiffness has hardly been studied and that this topic requires further investigation.

Last, it is noteworthy that the pit membranes of vestured pit species (Figure 1D) were considerably thinner than that of non-vestured pit species (Figure 1C) (all values were <300 nm, while non-vestured pit species had thickness values ranging from 300 to 600 nm). Yet, vestured pit species exhibited a considerable variation in leaf *P*_50_ including species with highly resistant xylem. This result reinforces the hypothesis that vestures play a role in preventing pit membrane stretching, increasing stiffness and thus embolism resistance. Further attention to this structure is therefore warranted. Altogether, this study provides additional evidence for the importance of interconduit pit membranes in determining embolism resistance not only in stems (Choat et al., 2008) but also in leaves and raises questions on how embolism propagates from embolized to sap-filled conduits in these organs.

*Conflict of interest statement*. The author has no conflicts of interest to declare.
